# G1 Interacts with OsMADS1 to Regulate the Development of the Sterile Lemma in Rice

**DOI:** 10.3390/plants13040505

**Published:** 2024-02-11

**Authors:** Huimin Fang, Hualan Chen, Jianing Wang, Ning Li, Long Zhang, Cunxu Wei

**Affiliations:** 1 Guangling College, Yangzhou University, Yangzhou 225000, China; hmfang@yzu.edu.cn (H.F.); w2669941371@163.com (J.W.); 2 Key Laboratory of Crop Genetics and Physiology of Jiangsu Province, Key Laboratory of Plant Functional Genomics of the Ministry of Education, Yangzhou University, Yangzhou 225009, China; chenhualan0210@163.com (H.C.); lining20222024@126.com (N.L.); zhanglong@yzu.edu.cn (L.Z.); 3 Co-Innovation Center for Modern Production Technology of Grain Crops of Jiangsu Province, Joint International Research Laboratory of Agriculture & Agri-Product Safety of the Ministry of Education, Yangzhou University, Yangzhou 225009, China

**Keywords:** ALOG, DUF640, empty glume, floral organ, protein interaction

## Abstract

Flower development, as the basis for plant seed development, is principally conserved in angiosperms. At present, a number of genes regulating flower organ differentiation have been identified, and an ABCDE model has also been proposed. In contrast, the mechanism that regulates the development of the sterile lemma remains unclear. In this study, we identified and characterized a rice floral organ mutant, M15, in which the sterile lemma transformed into a lemma-like organ. Positional cloning combined with a complementary experiment demonstrated that the mutant phenotype was restored by *LONG STERILE LEMMA1*/(*G1*). *G1* was expressed constitutively in various tissues, with the highest expression levels detected in the sterile lemma and young panicle. G1 is a nucleus-localized protein and functions as a homomer. Biochemical assays showed that G1 physically interacted with OsMADS1 both in vitro and in vivo. Interestingly, the expression of *G1* in M15 decreased, while the expression level of *OsMADS1* increased compared with the wild type. We demonstrate that G1 plays a key role in sterile lemma development through cooperating with OsMADS1. The above results have implications for further research on the molecular mechanisms underlying flower development and may have potential applications in crop improvement strategies.

## 1. Introduction

Flower development is one of the most significant events in plants’ transition from vegetative to reproductive growth. Based on analyses of flower mutants of *Antirrhinum majus* and *Arabidopsis thaliana*, the ABC model was proposed to explain how genes determine the identity of the floral meristem and control floral organ differentiation [1]. In recent years, this model has been further supplemented and developed into the ABCDE model and successfully applied to a variety of plants including rice [2,3,4,5].

**Figure 1:**
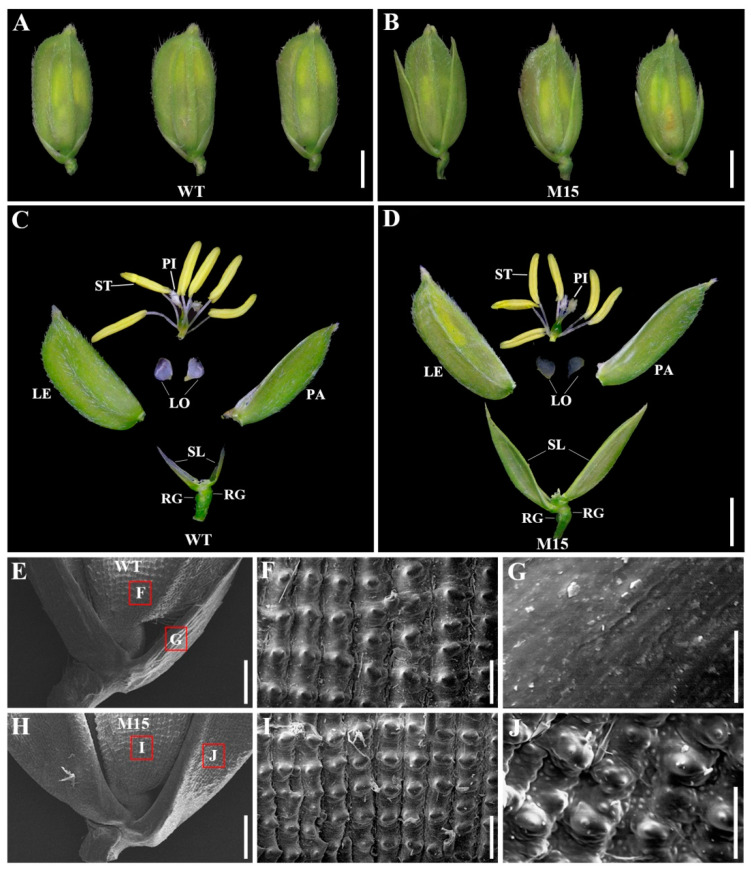
The spikelet structures of wild-type (WT) and mutant (M15) rice. (**A**–**D**) Phenotypes of WT (**A**,**C**) and M15 (**B**,**D**) spikelets at the heading stage. (**E**–**J**) Scanning electron microscope analysis of WT (**E**–**G**) and M15 (**H**–**J**) lemmas and sterile lemmas. RG: rudimentary glume; LE: lemma; LO: lodicule; PA: palea; PI: pistil; SL: sterile lemma; ST: stamen. Scale bars = 2 mm (**A**–**D**), 500 μm (**E**,**H**), 100 μm (**F**,**I**), and 50 μm (**G**,**J**).

Rice (*Oryza sativa*), as a staple food for more than half of the population, is one of the most important crops in the world. The development of rice floral organs is closely related to the number of grains per panicle and affects the rice yield. The inflorescence of rice is named a panicle, with a spikelet as its structural unit. A typical rice spikelet comprises one fertile floret, two empty glumes (also called sterile lemmas) and two rudimentary glumes, and the floret consists of one lemma, one palea, two lodicules, six stamens and one pistil (Figure 1A,C) [6]. Recently, a number of genes belonging to the ABCDE model that control the development of rice floral organs were identified and characterized in rice, most of which belong to the MADS box gene family [7].

Plant MADS box transcription factors are MIKC-type proteins, including highly conserved MADS (M) box DNA-binding domain, intervening (I) domain, keratin-like (K) domain and C-terminal (C) domain [8]. The rice A-function gene, *Degenerative palea/OsMADS15*, determines the characteristics of the lemma and palea [9]. *OsMADS2*, *OsMADS4* and *SUPERWOMAN1(SPW1)/OsMADS16* belong to B-class genes. Both OsMADS2 and OsMADS4 interact with OsMADS16 to regulate the development of the lodicule and stamen [10,11]. Two C-class genes, *OsMADS3* and *OsMADS58*, belong to the *AGAMOUS* (*AG*) subfamily. *OsMADS3* plays a key role in regulating stamen development, and *OsMADS58* mainly functions in the development of the carpel [12]. Rice *OsMADS13* and *OsMADS21*, as the homologs of Arabidopsis *SEEDSTICK* (*STK*) and *FLORAL BINDING PROTEIN* (*FBP*), are two D-class genes. *OsMADS13* regulates the ovule identity specification and also has a function in floral meristem determinacy [13,14]. *OsMADS21* has lost the ability to determine ovule identity during evolution [13]. To date, at least five E-class genes have been identified in rice, including *OsMADS1/LEAFY HULL STERILE1* (*LHS1*), *OsMADS5*, *OsMADS7*, *OsMADS8* and *OsMADS34/(PANICLE PHYTOMER2)PAP2* [15,16,17,18]. OsMADS1/LHS1 mainly specifies the identity of the lemma and palea and the development of the inner floral organ [19,20]. OsMADS7 and OsMADS8, two homologs of Arabidopsis SEPALLATA3 (SEP3), are shown to be involved in floral development redundantly [16]. OsMADS34 has versatile functions in lemma/palea, lodicule, stamen, carpel and spikelet development [21].

The rice floret is flanked by a pair of glume-like organs which are regarded as vestigial organs of two lower florets during the evolution of *Oryza*. The glume-like organs, which are called empty glumes or sterile lemmas in different publications in the rice literature, are not usually observed in the spikelets of maize and wheat [22]. The ectopic expression of rice *OsMADS1* causes spikelet alteration with elongated sterile lemmas [23]. The loss of function of *OsMADS34* leads to large sterile lemmas [24]. In addition to two E-class genes, *OsMADS1 and OsMADS34*, the *LONG STERILE LEMMA1*(*G1*)*/Elongated empty glume* (*ELE*) gene is also involved in the development of sterile lemmas [22,25]. *G1/ELE* encodes a protein with a domain of unknown function 640 (DUF640), and its mutation results in a long sterile lemma [22,25]. However, the mechanism of G1 controlling sterile lemma development remains poorly understood, and whether G1, OsMADS1 and OsMADS34 jointly regulate sterile lemmas remains to be identified.

In this study, as part of our continuous effort to understand the molecular machinery responsible for sterile lemma development in rice, we isolated a novel allele of *G1* which confers grain with long sterile lemma. Meanwhile, the functional characteristics of G1 including expression pattern, subcellular localization, and interacting proteins were characterized and identified. Our objective was to provide valuable information for understanding the functions of G1 and OsMADS1 in flower development and identities of sterile lemma.

## 2. Results

### 2.1. The M15 Mutant Shows Elongated Sterile Lemmas

In WT spikelets, the length of the sterile lemma was only one-quarter of the lemma/palea (Figure 1A). We obtained a ^60^Co-induced rice mutant, M15, which showed long and large sterile lemmas (Figure 1B). In most spikelets, the two sterile lemmas nearly reached the length of the lemma/palea. Except for the change in the sterile lemma, the lemma, palea, stamen, pistil and lodicule of the M15 mutant were not significantly different from those of the WT (Figure 1C,D), suggesting that the M15 mutation specifically affects the development of the sterile lemma. Furthermore, scanning electron microscopy was applied to compare the surface structures of the spikelet organs of WT and M15 rice (Figure 1E–J). The epidermal cells of the WT lemma were characterized with rows of round bulges tipped with sharp projections (Figure 1F), while the surface of the sterile lemma was smooth (Figure 1G). The surface morphology of the M15 lemma was largely comparable with the WT (Figure 1I), while the elongated sterile lemma of M15 was transformed into the lemma or palea, displaying a bulge similar to those in the lemma and palea (Figure 1J). These results collectively suggested that the sterile lemma in M15 is not only larger but also mimics the lemma. In addition, compared with the wild type, the grain length of M15 is significantly reduced, while there is no significant change in grain width or thickness (Figure S1).

### 2.2. Positional Cloning and Complementation of the Mutated Gene in M15

**Figure 2:**
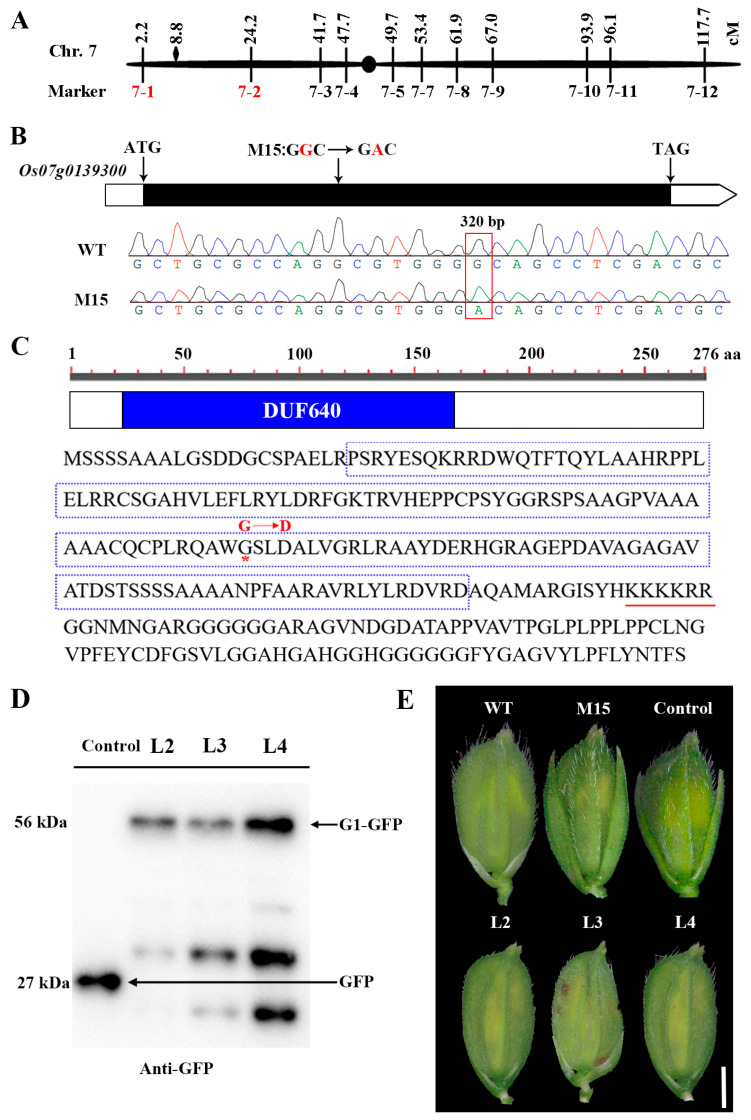
Identification of mutated gene and complementation of the M15 mutant. (**A**) The mutated gene was mapped to the short arm of chromosome 7 between markers 7-1 and 7-2. cM: centimorgan. (**B**) Mutation site and sequence chromatograms in *Os07g0139300*. (**C**) Amino acid sequence of G1. The DUF640 domain is marked with red font and the amino acids indicate the putative nucleus localization signal, which is underlined in red. The red asterisk represents that a single nucleotide substitution of guanine (G) to adenine (A) leads to a non-synonymous mutation from glycine (G) to aspartate (D). (**D**) Western blot analysis of G1-GFP fusion proteins from transgenic fragment-positive lines. The transgenic plants of free GFP were used as controls. L: line. (**E**) The functional complementation of the *G1* gene completely rescues the sterile lemma appearance. The phenotype of the sterile lemma in three positive transgenic lines was restored, while the control line still showed the phenotype of the M15 mutant. Scale bar = 2 mm.

To identify the mutant locus controlling the long sterile lemma phenotype, M15 was crossed with the indica variety Dular to produce an F_2_ mapping population. Twenty long sterile lemma plants and twenty normal individual plants were selected in the F_2_ population to generate two DNA pools. Then, 170 polymorphic insertion–deletion (InDel) markers on rice 12 chromosomes were applied to genotype the two pools for preliminary mapping. The gene was mapped to chromosome 7 between InDel markers 7-1 and 7-2, with genetic distances of 2.2 cM and 24.2 cM, respectively (Figure 2A). It has been reported that the *G1* gene regulates the development of glumes and is located at 8.8 cM of chromosome 7 [22], which is included in the markers of 7-1 and 7-2. Therefore, *G1* (*Os07g0139300*) is preferred as a candidate gene. Gene sequencing showed that the *G1* genomic sequences of M15 carried a single nucleotide substitution of guanine (G) to adenine (A) compared with the WT, leading to a non-synonymous mutation from glycine (G) to aspartic acid (D) at the 107th amino acid (Figure 2B,C). The *G1* gene contains only one exon and encodes a protein composed of 276 amino acid residues that harbors a DUF640 domain in the middle terminus (Figure 2C).

To test whether *Os07g0139300* was responsible for the M15 mutation, a p35S:G1-GFP recombinant plasmid was transferred into the M15 *calli*. The G1-GFP fusion protein was detected in positive transgenic lines using anti-GFP antibodies (Figure 2D). The phenotype of a sterile lemma in three positive transgenic lines was restored to the WT (Figure 2E), suggesting that G1-GFP was functional. In summary, the mutation of *G1* leads to the long sterile lemma of M15.

### 2.3. Expression Patterns of G1 and Subcellular Localization of G1

**Figure 3:**
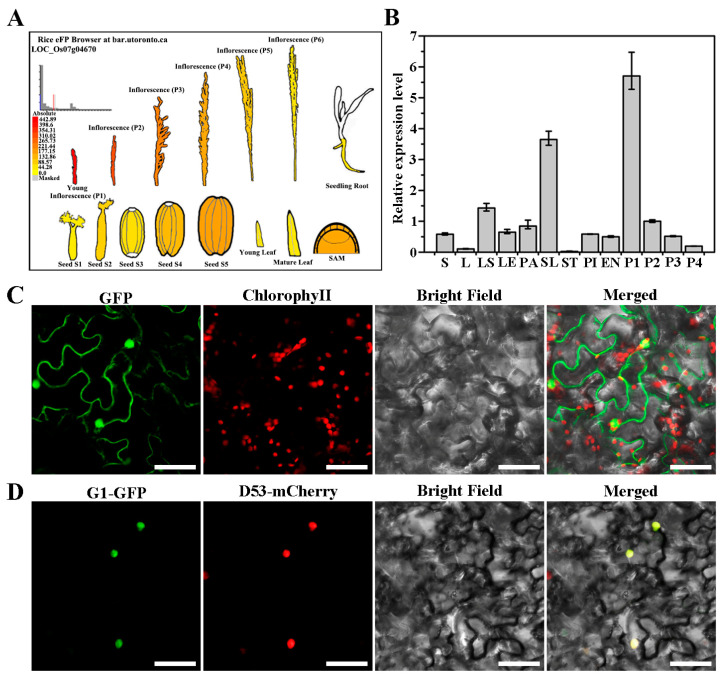
Spatiotemporal expression of G1 and subcellular localization of G1. (**A**) Expression patterns of G1 based on the rice eFP browser. (**B**) qRT-PCR analysis of G1 expression in various tissues. (**C**) Transient expression of free GFP in *N. benthamiana* leaves. (**D**) Transient expression of G1-GFP in *N. benthamiana* leaves. G1-GFP protein is located in the nuclei, overlapping with the nucleus marker D53-mCherry. GFP (green), chlorophyll autofluorescence (red) (C), mCherry (red) (D), bright-field images, and an overlay of green and red signals are shown. S: stem; L: leaf; LS: leaf sheath; LE: lemma, PA: palea, SL: sterile lemma, ST: stamen, PI: pistil. These tissues are taken from wild type plants just undergoing heading. EN: endosperm at 9 days after flowering, P1: 0.5~2 cm panicles, P2: 3~5 cm panicles, P3: 6 cm panicles, P4: panicles before heading. Data represent means and standard errors of three replicates. Scale bars = 50 μm (**C**,**D**).

*G1* was highly expressed in the early stage of spikelet development but was relatively low in seeds, leaves and roots, according to the Rice eFP Browser (http://bar.utoronto.ca/efprice/, accessed on 4 February 2024, Figure 3A). We then tested the *G1* expression pattern in various organs from the WT using a qRT-PCR. *G1* was expressed constitutively in all tested organs, including the stem, leaf, leaf sheath, lemma, palea, sterile lemma, stamen, pistil, endosperm and panicle, with the highest expression level detected in the sterile lemma and panicle (Figure 3B). Further, *G1* had the highest expression level during early panicle development, but this dropped dramatically as the spikelets developed from the P1 stage to the P4 stage (Figure 3B).

A WoLF PSORT [26] software analysis revealed that the G1 protein contains a nucleus localization signal (KKKKRR, Figure 2C). To determine the subcellular localization of G1, p35S:G1-GFP was expressed in *N. benthamiana* leaves. Free GFP was distributed evenly in the cytoplasm and nuclei, whereas G1-GFP fluorescence was detected only in the nuclei (Figure 3C) and co-localized with red signals from the nucleus marker D53-mCherry (Figure 3D). Therefore, G1 is a nucleus-localized protein.

### 2.4. G1 Protein Forms Homodimers

**Figure 4:**
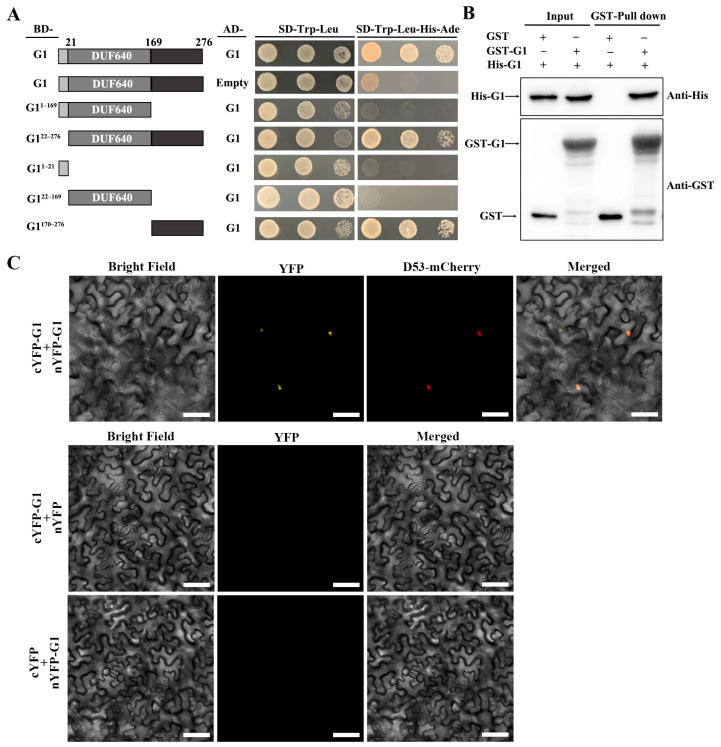
G1 forms a homocomplex. (**A**) Yeast two-hybrid assay showing the interaction between G1 and itself. (**B**) Pull-down assay showing the direct interaction between GST-G1 and His-G1 in vitro. (**C**) BiFC assay showing that G1 interacts with G1 in *N. benthamiana* leaf cells. Scale bars = 50 μm.

The G1 protein has transcription factor activity, according to the RiceData Browser (http://www.ricedata.cn, accessed on 4 February 2024). A transactivation assay was then performed using the full-length sequence and different truncations of G1 fused to the GAL4 DNA-binding domain in the Y2HGold yeast strain. Yeast transformants containing BD-G1, BD-G1^1–169^, BD-G1^22–276^, BD-G1^1–21^, BD-G1^22–169^ and BD-G1^170–276^ constructs grew well on an SD-Trp medium, whereas the growth of yeast transformants was completely inhibited on SD-Trp-His-Ade media (Figure S2). In summary, the G1 protein has no transactivation activity in yeast. Previous studies demonstrated that transcription factors can form homo- or heterodimers to function. A yeast two-hybrid assay showed that G1 can interact with itself (Figure 4A). Furthermore, the G1^170–276^ region (170–276 amino acid residues) in the C-terminus, rather than the N-terminus and DUF640, was required for the self-interaction of G1 (Figure 4A). A GST pull-down assay also confirmed self-interaction in vitro (Figure 4B). In addition, a BiFC analysis also showed that G1 can physically interact with itself in the nuclei of leaves from *N. benthamiana* (Figure 4C). In summary, we concluded that G1 is capable of forming homodimers.

### 2.5. G1 Interacts with OsMADS1

**Figure 5:**
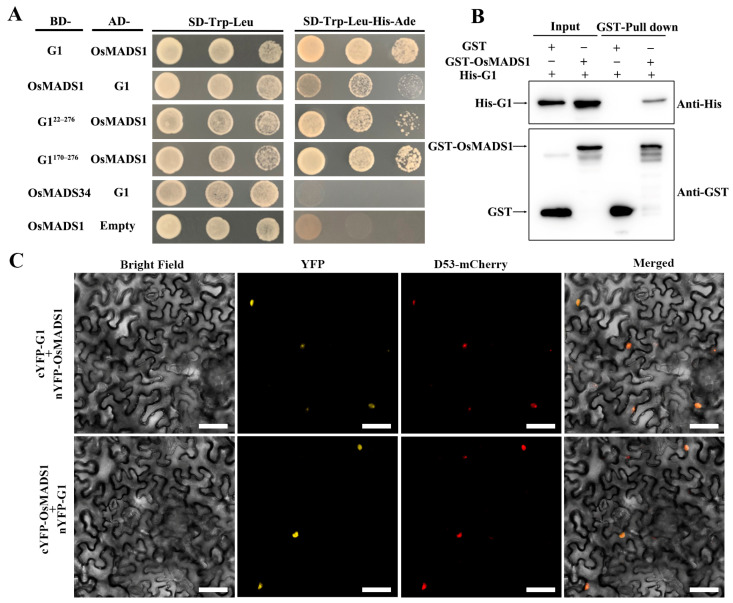
G1 interacts with OsMADS1. (**A**) Yeast two-hybrid assay showing the interaction between G1 and OsMADS1. (**B**) Pull-down assay showing the interaction between GST-OsMADS1 and His-G1 in vitro. (**C**) BiFC assay showing that G1 interacts with OsMADS1 in *N. benthamiana* leaf cells. Scale bars = 50 μm.

Previous studies demonstrated that OsMADS1 and OsMADS34 also participate in the development of sterile lemmas in rice [23,24]. In addition, earlier studies showed that OsMADS1 and OsMADS34 are localized to the nuclei [24,27]. Therefore, we speculated that G1, OsMADS1 and OsMADS34 interact with each other. To evaluate this possibility, we used the yeast two-hybrid assay and found that G1 and G1^170–276^ interact with OsMADS1 but not with OsMADS34 (Figure 5A). A pull-down assay was performed to confirm the physical interaction between G1 and OsMADS1 in vitro (Figure 5B). A BiFC experiment was performed to further confirm the interactions in vivo. In the BiFC assay, YFP fluorescence was produced in *N. benthamiana* leaves co-infected with *Agrobacterium* containing cYFP-G1 and nYFP-OsMADS1 or cYFP-OsMADS1 and nYFP-G1 (Figure 5C). These results indicated that G1 interacts with OsMADS1.

### 2.6. Expression Patterns of OsMADS1 and OsMADS34 in Rice

**Figure 6:**
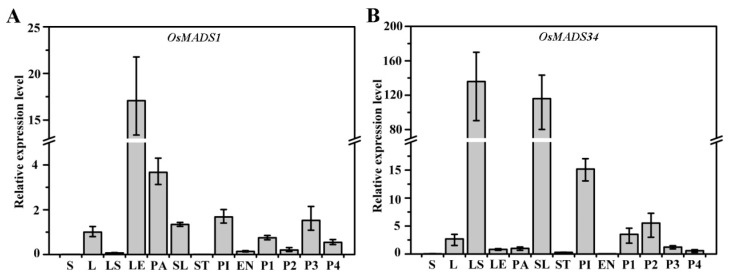
Expression patterns of *OsMADS1* and *OsMADS34* in rice. (**A**,**B**) Gene expression levels of *OsMADS1* (**A**) and *OsMADS34* (**B**) in various rice tissues. S: stem; L: leaf; LS: leaf sheath; LE: lemma, PA: palea, SL: sterile lemma, ST: stamen, PI: pistil. These tissues are taken from wild-type plants just undergoing heading. EN: endosperm at 9 days after flowering, P1: 0.5~2 cm panicles, P2: 3~5 cm panicles, P3: 6 cm panicles, P4: panicles before heading. Data represent means and standard errors (from at least three independent replicates).

To investigate the correlation between gene expression pattern and biological function, we examined the temporal and spatial expression patterns of *OsMADS1* and *OsMADS34*. As shown in Figure 6A, *OsMADS1* was highly expressed in the lemma, palea, sterile lemma, pistil and panicle, with low levels of expression in the stem, leaf sheath, stamen and endosperm. *OsMADS34* was highly expressed in the leaf sheath, sterile lemma, pistil and panicle, with low levels of expression in the stem, stamen and endosperm (Figure 6B). These results are consistent with the roles of *OsMADS1* and *OsMADS34* in rice sterile lemma development.

### 2.7. Loss-of-Function Mutation of G1 Altered the Expression of OsMADS1

**Figure 7:**
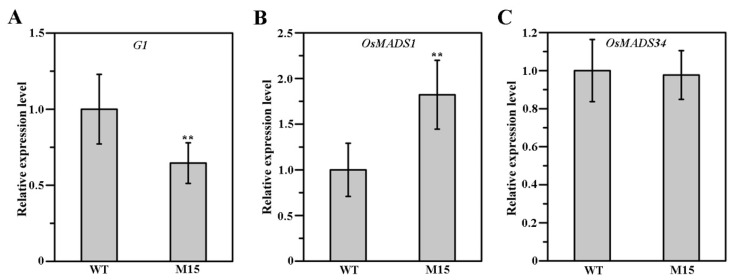
Expression levels of *G1* (**A**), *OsMADS1* (**B**) and *OsMADS34* (**C**) in the sterile lemmas of WT and M15. Data represent means and standard errors (from at least three independent replicates). Data were analyzed using a *t*-test; ** showed extremely significant difference (*p* < 0.01).

To explore the effect of G1 mutation on the expression of *G1*, *OsMADS1* and *OsMADS34*, the expression levels of three genes in the sterile lemmas of WT and M15 were analyzed using a qRT-PCR. The expression of *G1* in M15 was reduced by nearly half (Figure 7A), the expression of *OsMADS1* was significantly increased up to twofold (Figure 7B) and the expression of *OsMADS34* was almost unchanged compared with the WT (Figure 7C).

## 3. Discussion

### 3.1. G1 Controls the Specification of the Sterile Lemma

Rice spikelets are composed of one fertile floret, two sterile lemmas and two rudimentary glumes, and the sterile lemmas are located between the fertile floret and the rudimentary glumes. Some genes not only affect the development of the sterile lemma but also regulate the development of other flower organs. The AP2-family gene *SUPERNUMERARY BRACT* (*SNB*) regulates the transition from spikelet meristem to floral meristem. In *SNB* mutant plants, the sterile lemmas in some spikelets are transformed into lemma/palea-like organs, and the number of stamens and carpels is also altered [28]. In addition to affecting the number of sterile lemmas, mutations in *EXTRA GLUME1* (*EG1*) also lead to the formation of ectopic floral organs in each organ whorl [29]. In *nonstop glumes 1* (*nsg1*) mutants, the sterile lemma, palea, rudimentary glume and lodicule were elongated and transformed into lemma-like and/or a marginal region of the palea-like organs [30]. In this study, we isolated a long sterile lemma mutant, M15. Positional cloning and transgene complementation revealed that the mutant phenotype was controlled by *G1*. Except for the sterile lemma, the spikelet structure of M15 was complete, and the number and structure of other flower organs had not changed (Figure 1). Our SEM results further showed that the elongated sterile lemmas in M15 mimic lemmas (Figure 1). *G1* in M15 contained a missense mutation, and a substitution of G to A changed the glycine in the DUF640 domain to aspartate (Figure 2B,C). An amino acid sequence analysis showed that glycine (G) residue at amino acid position 107 was highly conserved in G1-related proteins among different plant species (Figure S3), suggesting that a single amino acid substitution in the DUF640 domain is critical for G1 protein function. Yoshida et al. [22] have reported four *G1* mutants with different mutations and named them *g1-1*, *g1–2*, *g1–3* and *g1–4*. The whole *G1* gene was deleted in *g1-1*, and missense and nonsense mutations were examined in *g1–2* (G82V, R117H), *g1–3* (G82V, A118V) and *g1–4* (68 bp deletion). *ele* is another *G1* mutant containing a missense mutation (G68D) [25]. Therefore, M15 is a new *G1/ELE* allelic mutant compared with the mutation sites that have been reported [22,25]. Though the mutations of these mutants are different, they all result in the same mutant phenotype, indicating that G1 plays an important and conservative role in the development of the sterile lemma. The ancestor of *Oryza* has three florets, and the three-floret spikelet hypothesis was confirmed using the *lateral florets 1* mutant [31]. Modern cultivated rice only bears a single fertile floret within a spikelet, and the two lateral florets degenerated and left only the lemma during evolution. Here we support the proposition that the rice sterile lemma belongs to a kind of lemma and palea and has the ability to transform into a lemma when G1 function is lost.

### 3.2. G1 and OsMADS1 Jointly Regulate Sterile Lemma Development in Rice

OsMADS1 plays a critical role in rice floral organ identity specification and floral meristem determinacy through interacting with B-, C- and D-class proteins. Previous studies show that OsMADS1 interacts with two A-class proteins, OsMADS14 and OsMADS15, and two E-class proteins, OsMADS7 and OsMADS8 [16,32]. In addition, OsMADS1 interacts with OsMADS3 and OsMADS58 to control floral meristem activity maintenance and regulate floral meristem determinacy, respectively [33]. In the present study, our yeast two-hybrid assay showed that G1 interacted with OsMADS1, and the interaction region was at the G1 C-terminus (Figure 5A). The BiFC assay revealed that G1 interacted with OsMADS1 in nuclei (Figure 5C). A temporal and spatial expression pattern analysis showed that both *G1* and *OsMADS1* were expressed abundantly in sterile lemmas and young panicles (Figure 3B and Figure 6A). In rice, the overexpression of *OsMADS1* results in an elongated sterile lemma [23]. Interestingly, the expression of *OsMADS1* also increased in the M15 sterile lemma (Figure 7A). In addition, the functional interaction between G1 and OsMADS1 is also strongly supported by phenotypic impacts on the sterile lemma. Although OsMADS34 is also involved in sterile lemma development [21,24], our yeast two-hybrid assay (Figure 5A) and qRT-PCR (Figure 7C) showed that there is no direct relationship between G1 and OsMADS34. These results imply that G1 and OsMADS1 play simultaneous roles in regulating the development of the sterile lemma.

### 3.3. Characteristics of G1

Domains of unknown function (DUFs) represent a large number of uncharacterized proteins [34]. Most DUFs are highly conserved in plants and are critical for plant growth and development. Rice and Arabidopsis contain 10 genes encoding proteins with DUF640, also named the ALOG (*Arabidopsis LSH1* and *Oryza G1*) domain [22]. The genes functionally identified in this family contain rice *BEAK-SHAPED GRAIN1* (*BSG1*), which is involved in controlling lemma and palea development. The loss of function of BSG1 leads to defects in lemma and palea expansion, but with a normal sterile lemma [35]. *G1* encoded a protein composed of a conserved DUF640 and specifically regulated the development of the sterile lemma (Figure 1 and Figure 2C). G1 is a homolog of rice BLS1 [35], while the changes in flower organs caused by the mutations of the two genes are completely different. Except for Arabidopsis, a BLASTP search showed that the homologs of G1 were found in other plants, including dicot and monocot plants (Figure S4). Moreover, a phylogenetic analysis indicated that these proteins were distinctly classified into two clades corresponding to proteins from monocots and dicots. Based on the functions of G1 homologs in Arabidopsis and rice, as well as the regulation of the sterile lemma and grain length in this study, we think that G1 is relatively conservative, while its roles in regulating plant development are diverse.

In this study, G1 was located in the nucleus and can form homodimers (Figure 3D and Figure 4). In addition, *G1* was highly expressed in the sterile lemma and the early developmental stage of the panicle (Figure 3B), implying that G1 may affect the growth of the sterile lemma by controlling the expression of other genes. Our qRT-PCR assay showed that the expression level of *OsMADS1* was significantly increased in the M15 mutant (Figure 7B). Previous studies have shown that overexpression of *OsMADS1* also leads to a long sterile lemma phenotype [23]. Therefore, we speculate that G1 may act as a suppressor of OsMADS1 to regulate sterile lemma development. Arabidopsis *LSH1*, a homolog of G1, has been reported to function as a transcription regulator protein [36]. The G1 protein structure was predicted using the I-TASSER database (https://zhanggroup.org/I-TASSER, accessed on 4 February 2024), and the predicted result was further analyzed using PyMOL software (2.5.7). The predicted ligand of G1 was a double-stranded nucleic acid, and the key amino acid sites of G1 binding to DNA are A105, V113, N153, P154, F155, G256, G257, G258, G260 and F261 (Figure S5). Collectively, these results support our interpretation that G1 is involved in transcriptional regulation for sterile lemma growth. In this study, we fused a GFP tag at the C terminal of G1 and obtained transgenic plants with G1-GFP. Therefore, in future work, we will use Chip-seq and IP-MS to identify the target genes and other interacting proteins of G1, respectively, to gain a more comprehensive understanding of the regulatory network involved in sterile lemma development.

## 4. Materials and Methods

### 4.1. Plant Materials

The long sterile lemma mutant M15 was derived from a mutant pool of the rice variety (*Oryza sativa* L. ssp. japonica) Kitaake (wild-type, WT) [37]. The mutant was self-crossed for over 5 generations with a stable phenotype and is considered homozygous. All rice materials were planted in the experimental field of Yangzhou University under natural conditions.

### 4.2. Microscopy

The developing spikelets of WT and M15 rice were separated using tweezers and an anatomical needle. The lemma, lodicule, palea, pistil, sterile lemma and stamen were photographed under a Leica EZ4W stereomicroscope. Mature and dry WT and M15 grains were glued onto aluminum specimen stubs to observe the lemma and sterile lemma using a Hitachi S-4800 scanning electron microscope (SEM), following the method described previously [37].

### 4.3. Positional Cloning and Transgene Complementation

The M15 mutant was crossed with a wide-compatibility indica variety Dular to obtain an F_2_ population. Twenty individuals with long sterile lemmas were selected from the F_2_ population for positional cloning. A bulked segregant analysis (BSA) was used to identify the markers linked to the M15 long sterile lemma phenotype [37].

For a complementation test, the *G1* coding sequence without a stop codon was cloned and inserted into vector pCAMBIA1305-GFP to produce the recombinant plasmid p35s:G1-GFP. p35s:G1-GFP and pCAMBIA1305-GFP were introduced into M15 *calli* using *Agrobacterium tumefaciens*-mediated transformation. Twenty-two independent transgenic lines harboring p35s:G1-GFP and 15 independent transgenic lines carrying pCAMBIA1305-GFP were successfully obtained. The transgenic plants of free GFP were used as a control.

### 4.4. RNA Isolation, cDNA Synthesis and Quantitative Real-Time PCR Analysis

Total RNA from different rice tissues was extracted using an RNAprep pure plant kit (Tiangen, Beijing, China) and reverse-transcribed to cDNA using a HiScript III 1st Strand cDNA Synthesis Kit (Vazyme, Nanjing, China). A quantitative real-time PCR (qRT-PCR) was performed on a CFX Connect real-time PCR system (Bio-Rad, Hercules, CA, USA) using the AceQ Universal SYBR qPCR Master Mix (Vazyme, Nanjing, China). The rice *ACTIN* gene was used as an endogenous control. The qRT-PCR primers are listed in Table S1.

### 4.5. Protein Extraction and Western Blot

Proteins were extracted in an extraction buffer (50 mM Tris-HCl (pH 8.0), 0.25 M Sucrose, 2 mM EDTA, 2 mM DTT, 1 mM PMSF) at 4 °C. The Western blot was prepared as described by Zhang et al. [37].

### 4.6. Subcellular Localization of G1

To determine the subcellular localization of G1, the empty plasmid pCAMBIA1305-GFP, p35s:D53-mCherry and p35s:G1-GFP were expressed in *Nicotiana. benthamiana* leaves using an *Agrobacterium tumefaciens*-mediated transformation. The infected tobacco leaves were cultured at 28 °C for 2–3 days, and the fluorescence signals were then observed using a Zeiss LSM880 confocal laser microscope.

### 4.7. Yeast Two-Hybrid Assay

The full-length coding sequences of *G1*, *OsMADS1* and *OsMADS34* and truncations of *G1* were cloned into a pGBKT7 vector. The coding sequences of *G1* and *OsMADS1* were cloned into a pGADT7 vector (Clontech, Palo Alto, CA, USA). Various combinations of plasmids were transformed into yeast AH109 competent cells according to the manufacturer’s instructions (Clontech, Palo Alto, CA, USA). The vectors pGBKT7 and pGADT7 were expressed in the yeast as negative controls. The PCR primers are listed in Supplementary Table S1.

### 4.8. Pull-Down Assay

The coding sequences of *G1* and *OsMADS1* were cloned into a pGEX4T-1 vector to generate fusion with glutathione S-transferase (GST), and the coding sequence of *G1* was then cloned into the pET-32a vector to generate fusion with a His tag. GST-G1, GST-OsMADS1, GST and His-G1 were transformed into BL21 Rosetta cells to induce protein expression using 0.5 mM isopropyl-β-d-thiogalactoside. The total protein concentration was quantified using a protein purification kit (Beyotime, Shanghai, China). The pull-down assay was performed as described previously [38]. The proteins were separated on a 10% SDS-PAGE gel and immunoblotted with anti-GST or anti-His antibodies (ABclonal, Wuhan, China).

### 4.9. Bimolecular Fluorescence Complementation (BiFC) Assay

The coding sequences of *G1* and *OsMADS1* were inserted into a pSAT1-cEYFP vector to create cYFP-G1 and cYFP-OsMADS1. The coding sequences of *G1* and *OsMADS1* were also inserted into a pSAT1-nEYFP vector to create nYFP-G1 and nYFP-OsMADS1. Various combinations of plasmids were then expressed in *Nicotiana. benthamiana* leaves as previously described [39]. Yellow fluorescent protein (YFP) fluorescence was observed using a Zeiss LSM880 confocal laser microscope. The relevant PCR primers are listed in Supplemental Table S1.

## 5. Conclusions

In this study, a new *G1* allelic mutant with long sterile lemma was identified. *G1* was highly expressed in the sterile lemma and young panicle, and its encoding protein was located in the nucleus. G1 functioned as a homomer and interacted with OsMADS1. The mutation of *G1* led to a decrease in its own expression level and an increase in the *OsMADS1* expression level. This study can provide useful information for better understanding the functions of *G1* and *OsMADS1* in flower development.

## Data Availability

Data are contained within the article and supplementary materials.

## Supplementary Materials

The following supporting information can be downloaded at https://www.mdpi.com/article/10.3390/plants13040505/s1, Figure S1: Phenotypes of WT and M15 seeds; Figure S2: The transcriptional activation assay of G1 and truncated G1 in yeast cells; Figure S3: Alignment of amino acid sequences of G1 protein mutation regions in different plant species; Figure S4: Phylogenetic analysis of G1 and its homologs; Figure S5: The protein structure analysis of G1; Table S1: Primers used in this study.
