# Supplementary material for: G1 Interacts with OsMADS1 to Regulate the Development of the Sterile Lemma in Rice
Source: Plants (Basel). 2024 Feb 11;13(4):505. doi: 10.3390/plants13040505 (PMC10892649; doi:10.3390/plants13040505)
Supplement: Supplementary file 1 [file plants-13-00505-s001.zip › Supplementary Figures S1-S5.pdf]

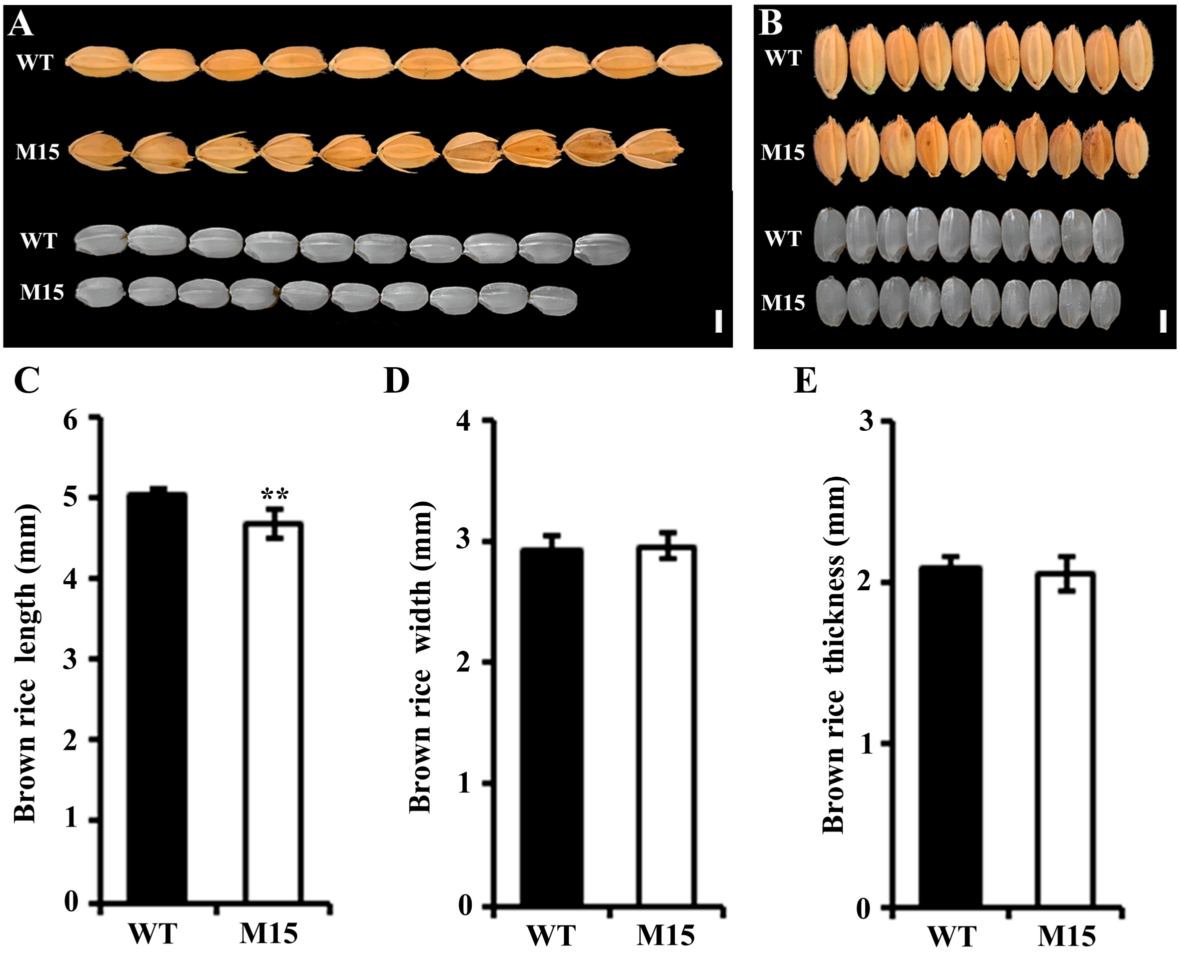

Supplemental Figure S1. Phenotypes of WT and M15 seeds. (A, B) Morphologies of grains (above) and brown rice (below) of WT and M15. (C-E): Quantification of brown rice length (C), width (D), and thickness (E) of WT and M15. Scale bars = 2 mm (A, B). Data represent means and standard errors (*n* = 20). Data analysed by t-test, ** showed extremely significant difference ($p < 0.01$).

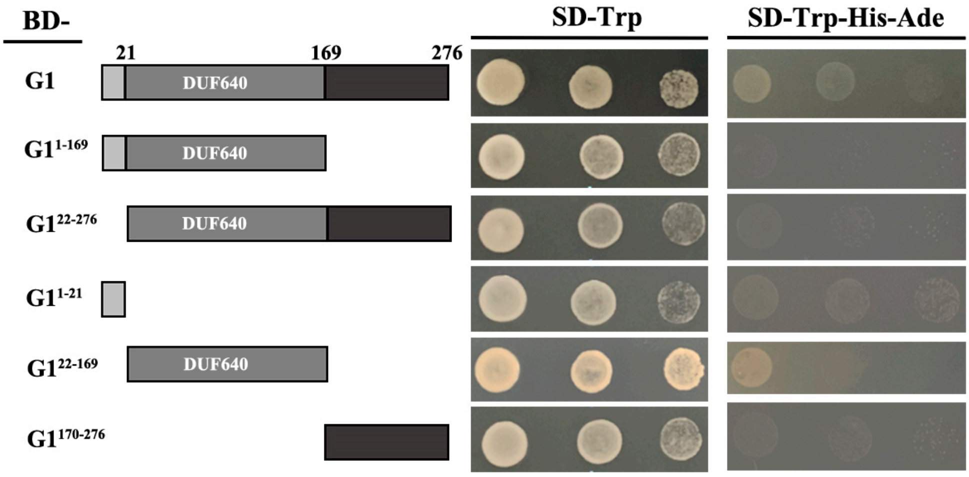

Supplemental Figure S2. The transcriptional activation assay of G1 and truncated G1 in yeast cells.

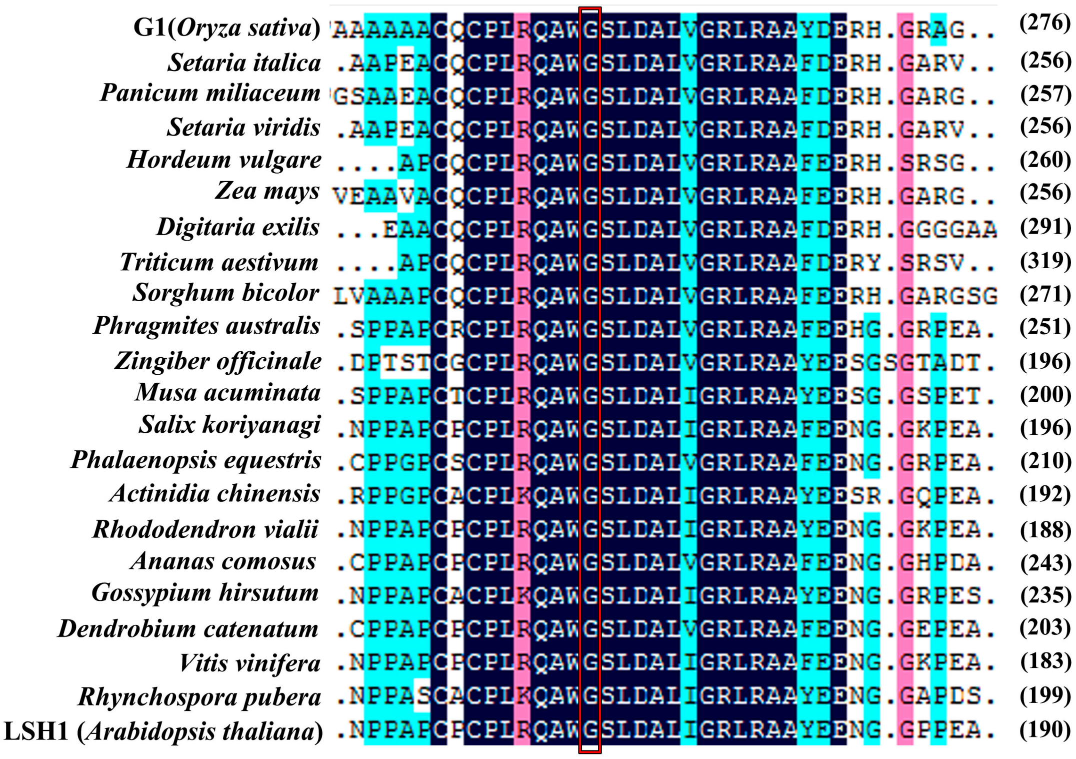

Supplemental Figure S3. Alignment of amino acid sequences of G1 protein mutation regions in different plant species. Highly conserved residues are highlighted with same color background. The amino acid sequence in the red box indicates the mutation in the protein of M15 mutant.

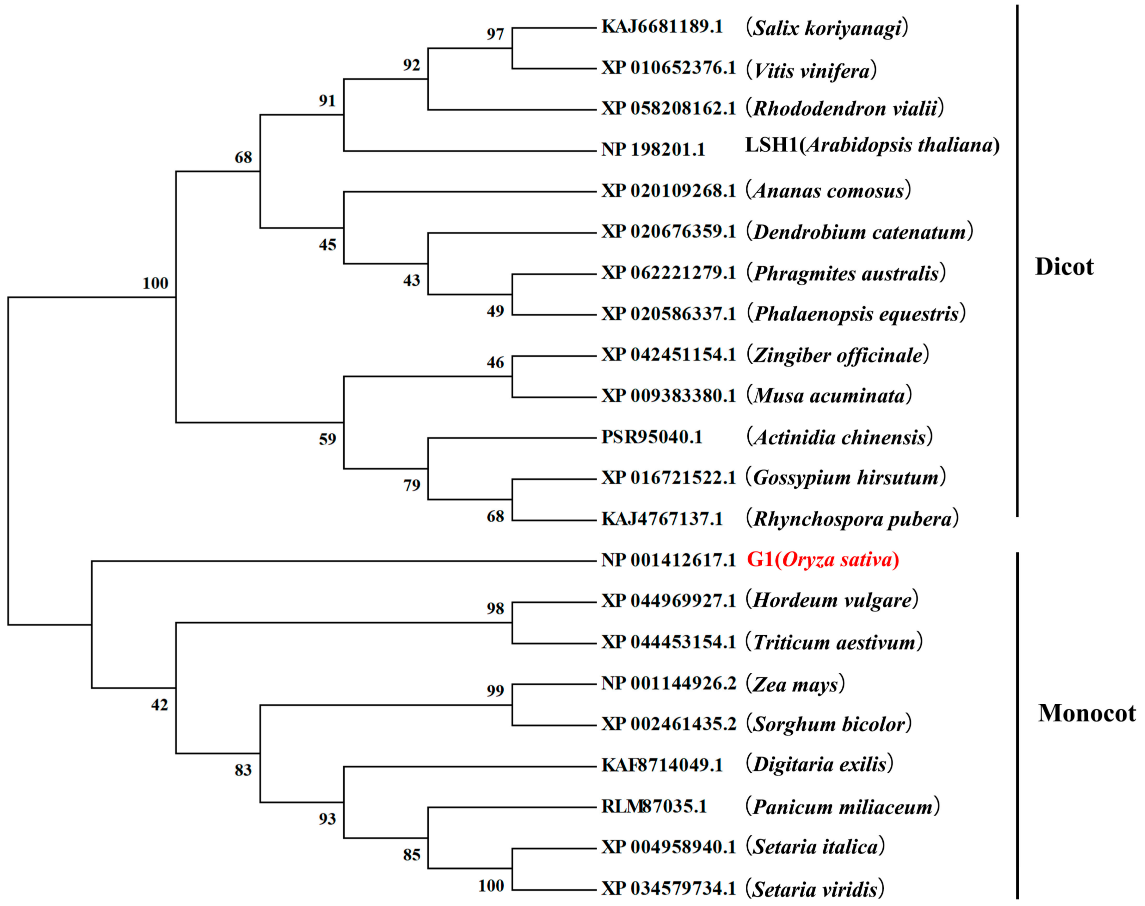

Supplemental Figure S4. Phylogenetic analysis of G1 and its homologs. The Neighbor-Join tree was constructed with MEGA5.0. The proteins are named according to their gene/EST names or NCBI accession numbers. Branch numbers represent a percentage of the bootstrap values in 1,000 sampling replicates.

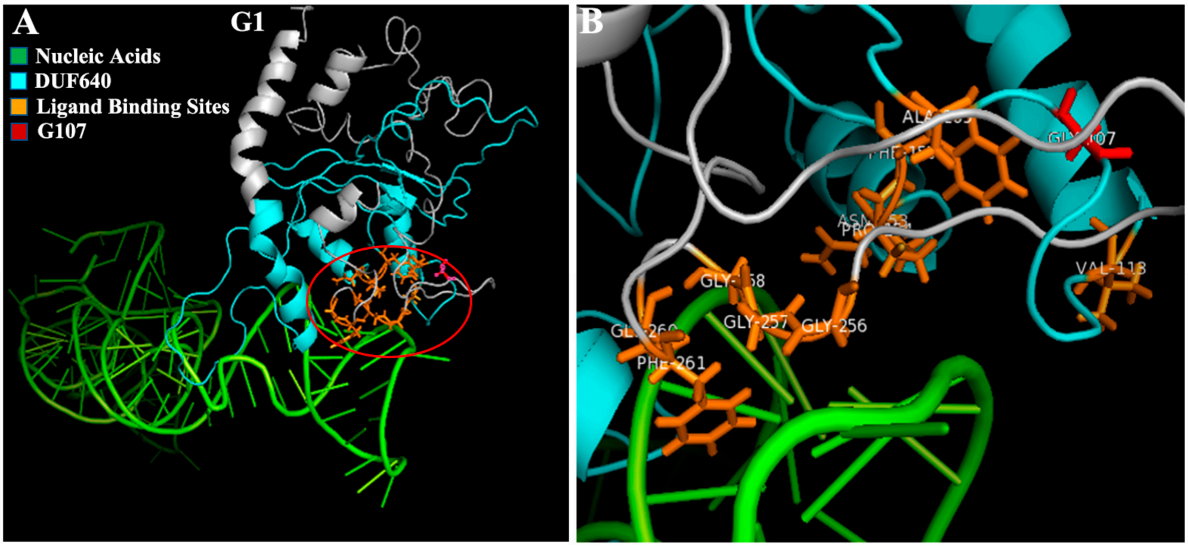

Supplemental Figure S5. The protein structure analysis of G1. (A): The prediction of G1 protein structure and ligand. (B): The key amino acid sites of G1 binding to DNA, including A105, V113, N153, P154, F155, G256, G257, G258, G260 and F261.
