# Supplementary material for: G1 Interacts with OsMADS1 to Regulate the Development of the Sterile Lemma in Rice
Source: Plants (Basel). 2024 Feb 11;13(4):505. doi: 10.3390/plants13040505 (PMC10892649; doi:10.3390/plants13040505)
Supplement: Supplementary file 1 [file plants-13-00505-s001.zip › Supplementary Tables S1.pdf]

Table S1. Primers used in this study.

Use	Primer Name	Sequence (5' to 3')
Complementation test	d35s: G1-GFP-F (SpeI)	ggacagcccagatcaactagtATGTCGTCGTCGTCCGCT
	d35s: G1-GFP-R (BamHI)	gcccttgctcaccatggatccACTGAAGGTGTTGTACAGAAATGGC
Quantitative RT-PCR	qRT-G1-F	CCGAGCCGGTACGAGTC
	qRT-G1-R	TCGAGGTAGCGGAGGAACT
	qRT-OsMADS1-F	GCTGCAACTACAACTCACAGG
	qRT-OsMADS1-R	TGATGGTGAGCATGAGGGTG
	qRT-OsMADS34-F	CCAACTAGCGATGAGATGCAGAAC
	qRT-OsMADS34-R	AGCCAGTGGAGCTAAATCCTCAC
	qRT-ACTIN-F	CCAAGGCCAATCGTGAGAAGA
	qRT-ACTIN-R	AATCAGTGAGATCACGCCCAG
Yeast two-hybrid	BD-G1-F (EcoRI)	atggccatggaggccgaattcATGTCGTCGTCGTCCGCTG
	BD-G1-R (BamHI)	ccgctgcaggtcgacggatccCTAACTGAAGGTGTTGTACAGAAAT
	BD-G1$^{1-169}$-F (EcoRI)	atggccatggaggccgaattcATGTCGTCGTCGTCCGCTG
	BD-G1$^{1-169}$-R (BamHI)	ccgctgcaggtcgacggatccGTCGCGGACGTCGCGCAGGT
	BD-G1$^{22-276}$-F (EcoRI)	atggccatggaggccgaattcCCGAGCCGGTACGAGTCGCAGAA
	BD-G1$^{22-276}$-R (BamHI)	ccgctgcaggtcgacggatccCTAACTGAAGGTGTTGTACAGAAAT
	BD-G1$^{1-21}$F (EcoRI)	atggccatggaggccgaattcATGTCGTCGTCGTCCGCTG
	BD-G1$^{1-21}$-R (BamHI)	ccgctgcaggtcgacggatccCCGCAGCTCCGCCGGCGAG
	BD-G1$^{22-169}$-F (EcoRI)	atggccatggaggccgaattcCCGAGCCGGTACGAGTCGCAGAA
	BD-G1$^{22-169}$-R (BamHI)	ccgctgcaggtcgacggatccGTCGCGGACGTCGCGCAGGT
	BD-G1$^{170-276}$-F (EcoRI)	atggccatggaggccgaattcGCGCAGGCCATGGCGCGC
	BD-G1$^{170-276}$-R (BamHI)	ccgctgcaggtcgacggatccCTAACTGAAGGTGTTGTAC
	BD-OsMADS1-F (EcoRI)	atggccatggaggccgaattcATGGGGAGGGGGAAGGTG
	BD-OsMADS1-R (BamHI)	ccgctgcaggtcgacggatccTCATATCCAGCCGGATGGG
	BD-OsMADS34-F (EcoRI)	atggccatggaggccgaattcATGGGGCGAGGCAAGGTG
	BD-OsMADS34-R (BamHI)	ccgctgcaggtcgacggatccCTAGGCCATCCACTCAGGA
	AD-G1-F (EcoRI)	gccatggaggccagtgaattcATGTCGTCGTCGTCCGCTG

	AD-G1-R (BamHI)	cagctcgagctcgatggatccCTAACTGAAGGTGTTGTACAGAAAT
	AD-OsMADS1-F (EcoRI)	gccatggaggccagtgaattcATGGGGAGGGGGAAGGTG
	AD-OsMADS1-R (BamHI)	cagctcgagctcgatggatccTCATATCCAGCCGGATGGG
BiFC assay	cYFP/nYFP-G1-F (SalI)	cttcgaattctgcagtcgacATGTCGTCGTCGTCCGCTGC
	cYFP/nYFP-G1-R (BamHI)	actctagatcaggtggatccCTAACTGAAGGTGTTGTACAGAAAT
	cYFP/nYFP-OsMADS1-F (SalI)	cttcgaattctgcagtcgacATGGGGAGGGGGAAGGTG
	cYFP/nYFP-OsMADS1-R (BamHI)	actctagatcaggtggatccTCATATCCAGCCGGATGGG
Pull-down assay	GST-G1-F (BamHI)	gatctggttccgcgtggatccATGTCGTCGTCGTCCGCTGC
	GST-G1-R (SalI)	gatgcggccgctcgagtcgacCTAACTGAAGGTGTTGTACAGAAAT
	His-G1-F (BamHI)	gccatggctgatatcggatccATGTCGTCGTCGTCCGCTGC
	His-G1-R (SalI)	tgcggccgcaagcttgtcgacCTAACTGAAGGTGTTGTACAGAAAT
	GST-OsMADS1-F (BamHI)	gatctggttccgcgtggatccATGGGGAGGGGGAAGGTG
	GST-OsMADS1-R (SalI)	gatgcggccgctcgagtcgacTCATATCCAGCCGGATGGG
